# Effect of a patient decision aid (PDA) for type 2 diabetes on knowledge, decisional self-efficacy, and decisional conflict

**DOI:** 10.1186/s12913-016-1262-4

**Published:** 2016-01-14

**Authors:** Robert. A. Bailey, Michael Pfeifer, Alicia C. Shillington, Qing Harshaw, Martha M. Funnell, Jeffrey VanWingen, Nanada Col

**Affiliations:** 1 Janssen Scientific Affairs, LLC, Raritan, NJ USA; 2 EPI-Q Inc, 1315 W 22nd Street, Suite 410, Oakbrook, IL 60523 USA; 3 Department of Learning Health Sciences, University of Michigan Medical School, Ann Arbor, MI USA; 4 Family Medicine Specialists, Grand Rapids, MI USA; 5 Five Islands Consulting, Georgetown, ME USA

**Keywords:** Type 2 diabetes mellitus, Antihyperglycemic medication, Shared decision-making, Patient decision aid, Decisional conflict, Decision self-efficacy

## Abstract

**Background:**

Patients with type 2 diabetes (T2DM) often have poor glycemic control on first-line pharmacologic therapy and require treatment intensification. Intensification decisions can be difficult because of many available options and their many benefits and risks. The American Diabetes Association recommends patient-centered, evidence-based tools supporting shared decision-making between patients and clinicians. We developed a patient decision aid (PDA) targeting decisions about treatment intensification for T2DM. Our objective was to determine the effectiveness of this PDA for patients with T2DM on metformin who require treatment intensification.

**Methods:**

This study was a pragmatic randomized controlled trial conducted in 27 US primary care and endocrinology clinics. Subjects were English-speaking adults with T2DM receiving metformin with persistent hyperglycemia who were recommended to consider medication intensification. Subjects were randomized to receive either the PDA or usual care (UC). Main outcome measures were change in knowledge, decisional self-efficacy, and decisional conflict.

**Results:**

Of 225 subjects enrolled, 114 were randomized to the PDA and 111 to UC. Mean [SD] age was 52 [1] years, time since T2DM diagnosis was 6 [+/−6] years, 45.3 % were male, and most (55.5 %) were non-Caucasian. Compared to UC, PDA users had significantly larger knowledge gains (35.0 % [22.3] vs 9.9 % [22.2]; *P* < 0.0001) and larger improvements in self-efficacy (3.7 [16.7] vs−3.9 [19.2]; *P* < 0.0001) and decisional conflict (−22.2 [20.6] vs−7.5 [16.6]; *P* < 0.0001).

**Conclusions:**

The PDA resulted in substantial and significant improvements in knowledge, decisional conflict and decisional self-efficacy. Decisional conflict scores after PDA use were within the range that correlates with effective decision-making. This PDA has the potential to facilitate shared-decision-making for patients with T2DM.

**Trial registration:**

NCT02110979

**Electronic supplementary material:**

The online version of this article (doi:10.1186/s12913-016-1262-4) contains supplementary material, which is available to authorized users.

## Background

Patients with type 2 diabetes mellitus (T2DM) with persistent hyperglycemia are at high risk of developing disease-related complications [2, 3]. Choosing therapy for patients when first-line therapy with metformin is no longer effective is complex and involves difficult trade-offs. Therapeutic options include adding an additional oral or injectable agent, including insulin [4]. A variety of antihyperglycemic medications are now available, each differing in important dimensions (e.g., effectiveness, side-effects, hypoglycemia risk, cost, impact on weight, and contraindications). There is no single best treatment appropriate for all patients, but rather the best treatment depends on what is important to each patient, their treatment goals, their underlying disease, and their comorbidities. Patients with diabetes often have inadequate information about their disease, their treatment options, and the consequences of their treatment decision, which can interfere with informed decision-making [5, 6]. Inadequate knowledge about medication and self-care in patients with diabetes negatively impacts self-efficacy, the belief that one is informed, supported, capable of taking steps and making decisions to improve health [7, 8]. Low self-efficacy is associated with lack of follow through on healthy intentions and decisions [9]. Inadequate knowledge and support also increase the likelihood of decisional conflict which is manifested by delays in decision-making, vacillating between choices, questioning values and tension [10]. Improving knowledge can promote diabetes self-efficacy, reduce decisional conflict, improve self-management behaviors [1, 11, 12], improve medication adherence [13, 14] and glycemic control [15–17], which should lead to improved clinical outcomes.

The American Diabetes Association (ADA) [18], and other medical organizations [19–22] recommend a patient-centered approach and patient engagement, including shared-decision making, to choosing among antihyperglycemic medications. They encourage clinicians to ensure that patients understand the decision and consider the risks and benefits involved in intensifying treatment (including the risk of hypoglycemia) [23, 24] and consider patient preferences. Helping patients make decisions aligned with their personal values is is integral to patient-centered care, an Institute of Medicine (IOM) mandate [25]. Yet implementing shared decision-making recommendations as a component of routine clinical care has been limited because of practice and health system barriers (e.g., limited time) and patient barriers (e.g., health literacy) [26]. Adoption of these recommendations will likely require targeted decision support tools to overcome these barriers.

Patient decision aids (PDAs) are educational tools designed to help patients make treatment decisions in collaboration with their clinician and promote shared decision-making. The Cochrane Collaboration systematic review of PDAs found they consistently improved knowledge, helped subjects match their values to their choices, and reduced passivity in decision-making [27]. PDAs focusing on patients with T2DM addressed statin choice [28–31] goal-setting [32, 33] metabolic control [34], addition of insulin [35], and starting or changing statins or antihyperglycemic treatment [36]. These PDAs promoted patients’ discussions of medications with their clinician, realistic expectations, autonomy, trust, engagement in decision-making, patient knowledge, risk perceptions, and documented goals, but none addressed decisional self-efficacy. Self-efficacy has been shown to be an important mediator of health decisions and health behaviors [37]. Additionally, none of these PDAs targeted the important decision about further treatment options that confronts the many patients with T2DM for whom first-line therapy with metformin is no longer effective and who need to consider further treatment options. This study was conducted in 27 non-academic sites across the country, giving much-needed evidence about the use of PDAs is real world environments.

The objective of this study was to determine the impact of a PDA for decisions about antihyperglycemic medications on key elements of shared decision-making (knowledge, self-efficacy, and decisional conflict) in a pragmatic randomized controlled trial involving a diverse national sample of subjects with T2DM during the course of routine clinical care.

## Methods

We conducted a multicenter randomized controlled pragmatic trial (Clinical Trials.gov Identifier: NCT02110979).

### Setting and participants

This trial was conducted in 27 primary care or endocrinology clinics throughout the US (Additional file 1). To improve the generalizability of the study, we restricted our national U.S. sample to non-academic clinical sites. Clinics were identified through a nationwide email list of providers maintained by the Primary Care Network™ (Springfield, MO), supplemented by providers’ emails obtained through a medical marketing database (IMD®). Clinicians were invited to participate and were selected if they were primary care clinicians or endocrinologists treating a minimum of ten patients with T2DM each week, had access to electronic medical records and/or electronic laboratory data, had a nurse, diabetes educator or other clinical staff support to facilitate subject identification, and were not affiliated with an academic institution. Approximately 2000 emailed invitations were sent; 68 sites responded; 32 were selected and 27 enrolled at least one subject.

Subject inclusion criteria: Eligible subjects were English-speaking adults with T2DM who were advised by their clinician to consider additional antihyperglycemic medication to a metformin-containing regimen to improve glycemic control. Subjects were further required to have a valid email address, access to the internet via a personal computer, and the ability to complete surveys online.

Subject exclusion criteria: Excluded were pregnant women, clinical trial participants, subjects already taking two or more medications in addition to metformin, or those with a lifetime exposure to more than three antihyperglycemic agents.

Patient Consent Procedures: All study procedures were approved by the New England IRB. Patient recruitment was intended to simulate how the PDA would be used in the ‘real world’ after trial completion. Potential study subjects were identified using medical records based on recent laboratory testing assessing glycemic control and rosters of appointments. Potential subjects were referred to the principle investigator by their clinician. Subjects were emailed a link to the study website (maintained by Qualtrics®) which screened for eligibility, obtained online consent, and collected baseline data. Subjects were randomized to either the online intervention (PDA group) or usual care (UC group). UC subjects were directed to follow-up with their doctor at an upcoming appointment as they normally would. Usual care was selected as the control arm in order to understand the incremental benefit of the PDA beyond care typically received during a clinical consultation and to make findings relevant to clinicians considering incorporating PDAs into routine practice. Referring clinicians were blinded to group assignment, unless they were incidentally un-blinded by subjects during a clinical consultation subsequent to enrollment (e.g., subjects mentioning the PDA or its contents during an office visit). Consistent with an intention-to-treat (ITT) principle [38, 39], we attempted to minimize loss to follow-up by monitoring subjects and prompting non-responders or partial responders to complete the intervention and/or assessments through phone and email reminders.

### The PDA

**Fig. 1:**
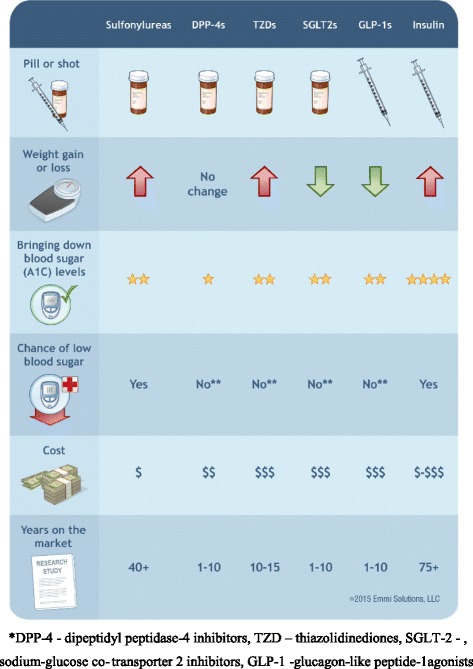
Type 2 Diabetes Medications Comparison Chart*. PDA “Fact Sheet”

The interactive Diabetes Decision Aid for T2DM targeted decisions about antihyperglycemic medication intensification for subjects for whom first-line treatment with metformin is no longer effective. The evidence-based PDA was designed to help subjects understand T2DM, its natural history, and the full range of treatment options, including sulfonylureas, dipeptidyl peptidase-4 (DPP-4 s) inhibitors, thiazolidinediones (TZDs), sodium-glucose co-transporter two (SGLT-2) inhibitors, glucagon-like peptide-1 (GLP-1 s) agonists, and insulin. To make the information more salient to subjects, evidence about the risks and benefits of different treatment options were organized and presented according to key preference domains that were identified during the development process [40] and through two focus groups with affected subjects. These domains include: 1) expected degree of glycemic control, 2) impact on weight, 3) risk of hypoglycemia and other adverse events, 4) route of administration, 5) frequency of dosing and route of administration and blood glucose monitoring, and 6) cost of therapy. The PDA described and compared the potential benefits and risks of the treatments in each of these domains and helped subjects explore their preferences and values through values clarification exercises [41]. It included questions eliciting their long-term goals for therapy and their concerns about antihyperglycemic medications. A summary “fact sheet” compared the risks and benefits of each class of medication using voice-over descriptions and simple graphics. Potential risks and benefits were illustrated using color-coded pictographs with plain language summaries, presented in a balanced, unbiased manner (Fig. 1).

The PDA did not allow subjects to skip through content in order to ensure that all key content areas were reviewed. It did not discuss lifestyle interventions (e.g., smoking, diet and exercise) but it did enable subjects to request information on these topics. The PDA was developed in accordance with the International Patient Decision Aid Standards criteria [42] in collaboration with a multidisciplinary team with expertise in clinical medicine, shared decision-making, and patient education [38].

### Intervention protocol

Prior to enrolling any subjects, clinicians and staff were encouraged to review the online PDA. Clinicians and clinic staff involved in identifying potential subjects participated in brief training sessions (30–60 min) on the use of the PDA and study procedures. Subjects randomized to the PDA group received computer-generated emails asking them to view information “recommended by their doctor” and a link to the PDA. Reminder messages were sent daily until the subject viewed the PDA in its entirety. Subjects who had not viewed the PDA within 5 days were contacted by phone or e-mail as a further reminder. The PDA took approximately 30 min to complete and subjects were encouraged to watch it as many times as they wanted and to make use of an online note-taking system to record questions or comments that could be shared with their doctor. Subjects were encouraged to view the PDA with others involved in the decision-making process.

### Outcomes and data collected

Study data were collected at baseline and 4–6 weeks following enrollment into the study for both study groups. In the PDA group, a brief survey was distributed shortly after viewing the PDA (within approximately 24 h) to assess opinions related to detail and length of content. A four to 6 weeks measurement time frame was selected in order to capture the impact of the PDA on knowledge gained, self- efficacy, and decisional conflict, outcomes which are best captured within the first month after exposure to the PDA.

Clinicians responded to similar questions. Subjects were paid $25 at each data collection point.

#### Baseline data

All subject data was self-reported and included socio-demographics (age, gender, race/ethnicity, educational level, employment and insurance status), duration of T2DM, height and weight, and relevant co-existing conditions (Table 1). Subjects were asked about their stage of decision-making [43], (e.g., had they begun to think about the decision, had they already made a decision), their preference for making clinical decisions, (e.g., do they prefer to have their clinician make decisions, prefer shared-decision-making or prefer making decisions on their own) [44] and the relative importance of competing values related to domains of diabetes medication decision-making. Subject values assessed included the importance of: managing blood sugar to a goal; taking a medication that might help lose weight or not cause weight gain; avoiding hypoglycemia (a “low”); avoiding side-effects such as pancreatitis, fractures, urinary tract infections and yeast infection; treatment costs, avoiding injections; and dosing convenience (i.e., taking medication more than once a day). These were assessed using a scale from 0 to 10, with 0 representing not at all important, and ten representing extremely important.

**Table 1:** Baseline characteristics

	PDA (*n* = 114)	Usual Care (*n* = 111)	*p*-value^a^
Mean age (Mean, SD)	53.0 (13.8)	51.6 (11.5)	0.4121
Male (n, %)	52 (45.6)	50 (45.1)	0.9317
Race/Ethnicity (n, %)			
White/Caucasian	53 (46.5)	48 (43.2)	0.6243
Black/African American	32 (28.1)	31 (27.9)	0.9810
Hispanic	14 (12.3)	15 (13.5)	0.8437
Asian/Pacific Islander	5 (4.4)	9 (8.1)	0.2810
Native American or Alaska Native	1 (0.9)	2 (1.8)	0.6183
Other or Multi-Racial	9 (7.9)	6 (5.4)	0.4542
Education (n, %)			
Grade School	2 (1.8)	1 (0.9)	0.5786
Some High School	12 (10.5)	2 (1.8)	0.0068
High School Graduate	29 (25.4)	29 (26.1)	0.9062
Some College	30 (26.3)	44 (39.6)	0.0334
College Graduate	26 (22.8)	24 (21.6)	0.8307
Graduate School	15 (13.2)	11 (9.9)	0.4461
Employment (n, %)			
Full time	50 (43.9)	60 (54.1)	0.3379
Part time	11 (9.6)	7 (6.3)	
Unemployed	15 (13.2)	11 (9.9)	
On Disability	19 (16.7)	11 (9.9)	
Student	2 (1.8)	2 (1.8)	
Homemaker	6 (5.3)	3 (2.7)	
Other	11 (9.6)	17 (15.3)	
Insurance status (n, %)			
PPO	39 (34.2)	49 (44.1)	0.2585
HMO	26 (22.8)	14 (12.6)	
Medicare	19 (16.7)	22 (19.8)	
Medicaid	16 (14.0)	10 (9.0)	
None	6 (5.3)	6 (5.4)	
Other	8 (7.0)	10 (9.0)	
Years since T2DM Diagnosis (Mean, SD)	6.8 (6.0)	6.5 (5.4)	0.7192
BMI (Mean, SD)	34.4 (8.0)	35.6 (9.4)	0.3152
Underweight (<18.5) (n,%)	0	0	0.8106
Normal (18.5–24.99)	13 (11.4)	11 (9.9)	
Overweight (25–29.99)	22 (19.3)	21 (18.9)	
Obese class I (30–34.99)	32 (28.1)	27 (24.3)	
Obese class II (35–39.99)	15 (13.1)	21 (18.9)	
Obese class III (>40)	32 (28.1)	31 (27.9)	
Comorbidities and Health Conditions (n,%)			
High Blood Pressure	82 (71.9)	69 (62.2)	0.1190
Heart Disease	10 (8.8)	9 (8.1)	0.8579
Stroke	3 (2.6)	5 (4.5)	0.4951
High Cholesterol	59 (51.8)	57 (51.4)	0.9518
High Triglycerides	23 (20.2)	27 (24.3)	0.4542
Gallbladder Disease	8 (7.0)	5 (4.5)	0.4192
Liver Disease	2 (1.8)	2 (1.8)	1.000
Kidney Disease or Renal Failure	1 (0.9)	2 (1.8)	0.6183
Pancreatitis	3 (2.6)	5 (4.5)	0.4951
Insulin Resistance	5 (4.4)	7 (6.3)	0.5216
Osteoporosis	3 (2.6)	2 (1.8)	1.000
Peripheral Vascular Disease	5 (4.4)	5 (4.5)	1.000
Hypoglycemia	3 (2.6)	5 (4.5)	0.4951
Ketoacidosis	0	2 (1.8)	0.2423
Neuropathy	12 (10.5)	9 (8.1)	0.5330
Yeast or Urinary Tract Infection	22 (19.3)	11 (9.9)	0.0466
Baseline Knowledge, Decisional Conflict and Decision Self-efficacy			
Knowledge (% correct, mean, SD)	20.4 % (15.7)	22.6 % (16.9)	0.3150
Knowledge confidence score (mean, SD)	56.1 (5.6)	56.9 (8.6)	0.4035
Decisional Conflict Scale (DCS) (mean, SD)	45.8 (18.8)	45.4 (16.9)	0.8559
Uncertainty subscale	49.9 (24.5)	50.8 (24.9)	0.7915
Informed subscale	56.9 (23.7)	58.5 (24.2)	0.6214
Support subscale	36.6 (19.7)	37.2 (18.4)	0.8422
Values Clarity subscale	48.6 (23.2)	45.1 (22.9)	0.2524
Effective Decision subscale	39.1 (19.5)	37.8 (16.5)	0.5880
Decisional Self-Efficacy Scale (DSES) (mean, SD)	85.9 (15.6)	85.6 (16.1)	0.8738

^a^numerical variables t-test; categorical variables: X^2^ or Fisher’s exact test

#### Primary outcome

The primary outcome of this study was knowledge gained about medication, using a scale developed for the study to assess understanding of how different treatments differ in terms of their 1) impact on glycemic control (amount and durability), 2) impact on weight, 3) risk of hypoglycemia and other adverse events, 4) route of administration, 5) frequency of dose administration and blood glucose monitoring, and 6) financial costs. Knowledge was assessed by asking subjects to respond to 17 statements about antihyperglycemic medications that were either true or false (Additional file 1). To discourage guessing, we offered a “not sure” response option. We calculated the percentage of correct answers. We also asked subjects to rate their confidence in their responses using a ten point Likert scale (from 1, “I am certain this is incorrect” to ten, “I am certain this is correct”, with five corresponding to “not sure”). All questions were pretested on ten subjects with T2DM and revised accordingly. The pretest suggested uniform comprehension of all but one item, which was subsequently reworded. A composite “perceived knowledge confidence” score was summed and normalized to a 0–100 scale. Knowledge gained was calculated by subtracting baseline from follow-up scores.

#### Secondary outcomes

Secondary outcomes included changes in the Decision Self Efficacy Scale (DSES) and Decisional Conflict Scale (DCS). The DSES assesses subjects’ self-confidence or belief in one’s abilities in decision-making. In testing, the DSES internal reliability coefficient was .92, and the scale significantly discriminated between subjects who did and did not make health-related decisions [45]. The DSES is summed and scored from 0 to 100 with 100 representing complete self-efficacy and 0 representing complete lack of self-efficacy. The DCS [46] measures overall uncertainty in making a health-related decision and includes subscales that address the factors that contribute to uncertainty (feeling uncertain, informed, clear about values, supported in effective decision-making). In validation testing, the test-retest reliability coefficient of the DCS was 0.81. Internal consistency coefficients ranged from 0.78 to 0.92. The DCS discriminated significantly between those who had strong intentions for and ultimately acted upon decision making and those whose intentions were uncertain [10]. The DCS and subscales are scored from 0 to 100; lower scores correspond to less decisional conflict [47]. Changes in DSES and DCS were calculated by subtracting baseline from follow-up scores.

### Data analyses

All subjects were analyzed in the arm to which they were randomized (ITT). Baseline demographic, past medical history and responses to baseline decision-related questionnaires were compared between treatment groups in univariate analyses to assess the success of randomization, using t-tests for continuous and X^2^ or Fisher’s exact for categorical data. For the primary and secondary outcomes, unadjusted mean differences in knowledge, DSES and DCS scores were calculated by subtracting post from baseline scores and comparing those values using t-tests. Covariate adjusted analysis on primary and secondary outcome were conducted using generalized linear models (GLM). Dependent variables in the models were knowledge gained, knowledge confidence score, DCS and DSES. Covariates included in the models were those of theoretical interest to the investigators (e.g. stage of decision-making, race/ethnicity) and those relating to failed randomization, using a threshold of p <0.1.

Adherence to the intervention group (viewing the PDA) was included in GLM models as a dichotomous variable (all or none), based upon tracking each participant’s unique subject code for accessing the PDA. UC controls did not have access to PDA; crossovers from the control to intervention group were not possible.

We assessed level of missing data for our key constructs and outcomes. No variables had >30 % missing thus we imputed missing data using a monotone data Markov-Chain-Monte-Carlo (MCMC) method (SAS/STAT procedure MIANALYZER) [48]. Analyses were computed using SAS (SAS Institute, Inc, Cary, NC).

### Data collection, quality and integrity

The survey software system (Qualtrics®) that was used to enroll, screen, and implement surveys has a variety of features to ensure data integrity. The survey questionnaires could only be completed by the person to whom links were sent, IP addresses were independently assessed to verify no unauthorized access, and data were written directly to the secure Qualtrics® database, which could not reviewed or changed once submitted by subjects as complete. All staff analyzing data were blinded to treatment group assignment. Referring clinicians were blinded to group assignment, unless they were incidentally un-blinded by subjects during a clinical consultation subsequent to enrollment (e.g., subjects mentioning the PDA or its contents during an office visit).

### Power analyses

Our study was powered to measure the impact of the PDA on the primary outcome of knowledge, using a 2-tailed test. In a Cochrane systematic review of 42 studies (10842 participants) measuring the impact of PDA vs usual care on knowledge the effect size was 13.34 [11.17, 15.51] [26]. Our study with a sample size of 110 in each of two arms was projected to have a 90 % chance to detect a difference of 13.34 or greater in knowledge gained.

## Results

**Fig. 2:**
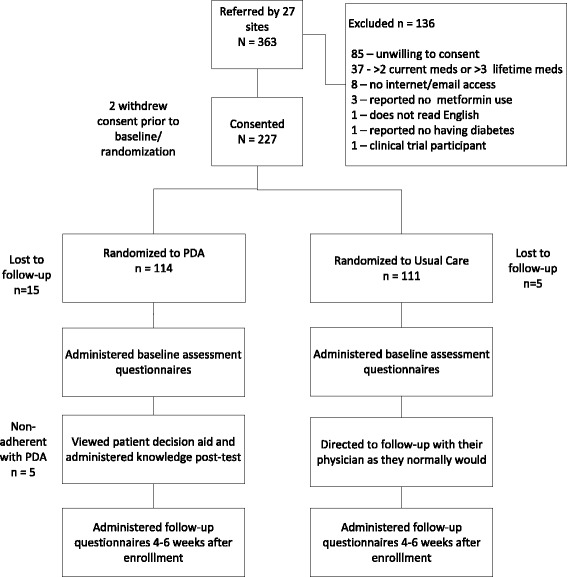
Subject Disposition

We randomized 225 subjects into the study (Fig. 2), 114 to the PDA and 111 to usual care. All subjects were followed for approximately 6 weeks after randomization except for 20 who were lost to follow-up (PDA group, *n* = 15; usual care group, *n* = 5). Twenty seven (27) clinicians enrolled at least 1 subject, with 15 enrolling more than five subjects.

Baseline demographic and clinical characteristics are presented in Table 1. The mean age of all subjects was 52.3 [sd 12.7] years, the majority were female (54.7 %) and non-Caucasian (55.5 %). Mean duration since T2DM diagnosis was 6.6 [sd 5.7] years. Baseline demographics and comorbidities were similar across both groups, with two exceptions. Fewer subjects in the PDA versus UC group reported having some college education (26.3 % vs 39.6 %, respectively), and fewer had a history of a yeast or urinary tract infection (UTI). Most (57.3 %) had not begun considering their choices for medications; most (71.1 %) preferred an active role in health care decision-making, either shared with their physician, or independently (Table 2).

**Table 2:** Baseline decisional characteristics

	PDA	Usual Care	*p*-value^a^
	*n* = 114	*n* = 111	
Stage of Decision-making	n (%)	n (%)	
Haven’t begun to think about the choices	37 (32.5)	31 (27.9)	0.4596
Haven’t begun to think about the choices, but am interested in doing so	30 (26.3)	31 (27.9)	0.7856
...are considering the options now	18 (15.8)	26 (23.4)	0.1489
...are close to selecting an option	3 (2.6)	1 (0.9)	0.6218
Have already made a decision, but am still willing to reconsider	15 (13.2)	14 (12.6)	0.9029
Have already made a decision and am unlikely to change my mind	11 (9.7)	8 (7.2)	0.5101
Control Preference			
Doctor-oriented	34 (29.8)	31 (27.9)	0.7537
Shared	51 (44.7)	58 (52.3)	0.2594
Independent	29 (25.4)	22 (19.8)	0.3142

^a^X^2^ test or Fisher exact test

**Fig. 3:**
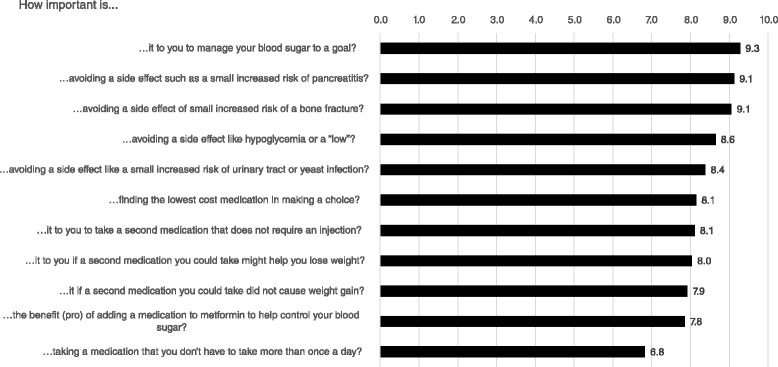
Subject Values Scores At Baseline for Outcomes Related to Treatment

Figure 3 compares the importance that participants placed on different outcomes affected by treatment (‘subject values’). There were no differences between the study groups so both groups were combined. The most important values were a medication’s ability to manage blood sugar to a goal, followed closely by avoidance of side-effects. Least important were the convenience of dosing.

### Knowledge outcomes

Subjects in the PDA group gained substantially more knowledge than those in the UC group at 6 week follow-up (35.0 % versus 9.9 % improvement in scores, respectively, *p* < 0.0001). (Table 3) This corresponds to subjects in the PDA group correctly answering nine of 17 knowledge questions regarding medication treatments. Knowledge confidence also improved substantially more in the PDA group versus UC (11.0 [sd 12.8] versus 1.6 [sd 8.9] respectively (*p* < 0.0001). In both groups, knowledge confidence score was correlated with knowledge, *r* = 0.476 (*p* < 0.001).

**Table 3:** Change in Scores between Baseline and Final Follow-up

	PDA	Usual Care	*p*-value^a^
	*n* = 114	*n* = 111	
	(Mean Δ, SD)	(Mean Δ, SD)	
Knowledge (% correct)	35.0 % (22.3)	9.9 % (22.2)	<.0001
Knowledge confidence	11.0 (12.8)	1.6 (8.9)	<.0001
Decision Self-Efficacy scale	3.7 (16.7)	−3.9 (19.2)	0.0018
Decisional Conflict Score (total)^b^	−22.2 (20.6)	−7.5 (16.6)	<.0001
Uncertainty subscale	−21.3 (27.3)	−9.5 (23.5)	0.0006
Informed subscale	−29.9 (26.5)	−8.4 (27.4)	<.0001
Values Clarity subscale	−27.1 (24.4)	−8.9 (22.1)	<.0001
Support subscale	−19.1 (20.7)	−5.8 (17.1)	<.0001
Effective Decision subscale	−15.7 (22.1)	−5.4 (18.6)	<.0001

^a^t-test

^b^Lower is considered better

In multivariate analyses that adjusted for gender, educational level, race, stage of decision-making, importance of cost in making medication decisions, comorbidities, and other factors, PDA use (versus usual care) was associated with a greater likelihood of knowledge score gains, 26.6 percentage points (*p* < 0.0001) and a 10.1 points (*p* < 0.0001) gain in knowledge confidence (Table 4).

**Table 4:** Adjusted analyses – primary and secondary outcomes. Generalized linear model regression model -primary and secondary outcomes

	Percent Correct Response Δ		Knowledge Confidence Δ		Decisional Conflict Δ		Decisional Self Efficacy Δ	
	Coefficient (SE)	*P* value, 5 % CI	Coefficient (SE)	P value, 95 % CI	Coefficient (SE)	*P* value, 95 % CI	Coefficient (SE)	*P* value, 95 % CI
PDA Group	26.60 (3.23)	<.0001 (20.23, 32.96)	10.13 ( 1.54)	<.0001 (7.10, 13.17)	−16.25 (2.57)	<.0001 (−1.33, −11. 18)	7.44 (2.56)	0.004 (2.39, 12.49)
Male	6.74 (3.26)	0.040 (0.31, 13.18)	1.27 (1.55)	0.414 (−1.79, 4.34)	1.78 (2.60)	0.494 (−3.34, 6.91)	0.67 (2.59)	0.796 (−4.43, 5.77)
Non-adherence with PDA	−0.50 (6.77)	0.941 (−13.85, 12.85)	−5.57 (3.23)	0.086 (−11.93, 0.79)	8.02 (5.39)	0.139 (−2.62, 18.65)	−6.65 (5.37)	0.217 (−17.24, 3.94)
Education – Grade School*	−4.34 (14.27)	0.762 (−32.48, 23.80)	−6.92 (6.80)	0.310 (−20.33, 6.49)	−28.07 (11.37)	0.014 (−50.49, −5.65)	20.28 (11.32)	0.075 (−2.04, 42.60)
Education – Some High School*	−5.31 (8.07)	0.512 (−21.22, 10.61)	−6.14 (3.85)	0.112 (−13.72, 1.44)	7.39 (6.43)	0.252 (−5.29, 20.06)	−3.72 (6.40)	0.562 (−16.34, 8.90)
Education – High School Graduate*	1.55 (5.61)	0.782 (−9.51, 12.62)	−5.88 (2.55)	0.002 (−13.78, 3.24)	3.81 (4.47)	0.396 (−5.01, 12.62)	−2.90 (4.45)	0.516 (−11.68, 5.88)
Education – Some College*	4.98 (5.36)	0.354 (−5.58, 15.54)	−7.31 (2.67)	0.022 (−10.92, −0.85)	−1.56 (4.27)	0.716 (−9.97, 6.85)	−6.05 (4.25)	0.156 (−14.42, 2.33)
Education – College Graduate*	2.67 (5.61)	0.635 (−8.40, 13.73)	3.78 (2.85)	0.007 (−12.58, −2.04)	2.11 (4.47)	0.638 (−6.71, 10.92)	−5.57 (4.45)	0.212 (−14.35, 3.20)
Stage of Decision-making† - Have not begun to think about choice	−2.03 (5.99)	0.735 (−13.83, 9.77)	−7.31 (2.67)	0.186 (−1.84, 9.41)	−12.86 (4.77)	0.008 (−22.26, −3.46)	3.90 (4.75)	0.413 (−5.46, 13.26)
Stage of Decision-making^†^- Have not begun to think about choice, but interested	1.36 (6.15)	0.825 (−10.76, 13.49)	6.71 (2.93)	0.023 (0.93, 12.49)	−11.78 (4.90)	0.017 (−21.44, −2.12)	6.7 1 (4.88)	0.171 (−2.91, 16.33)
Stage of Decision-making^†^- Considering options/close to selecting	1.05 (6.40)	0.870 (−11.57, 13.67)	5.75 (3.05)	0.061 (−0.27, 1 1.76)	−14.64 (5.10)	0.005 (−24.7, −4.58)	2.34(5. 08)	0.645 (−7.67, 12.35)
Stage of Decision-making^†^-already made decision, but willing to consider	0.54 (6.83)	0.938 (−12.94, 14.01)	5.68 (3.26)	0.083 (−0.74, 12.10)	−7.37 (5.44)	0.177 (− 18.11, 3.36)	6.37 (5.42)	0.241 (−4.32, 17.06)
Previous yeast or UTI History	−0.82 (4.62)	0.860 (−9.92, 8.28)	1.80 (2.20)	0.414 (−2.54, 6.14)	−3.24 (3.68)	0.379 (−10.49, 4.01)	−0.42 (3.66)	0.909 (−7.64, 6.80)
Values -finding lowest cost option	−0.14 (0.64)	0.825 (−1.40, 1.12)	−0.06 (0.30)	0.840 {−0.66, 0.54)	−0.20 (0.51 )	0.698 (−1.20, 0.81)	0.67 {0.51)	0.190 (−0.33, 1.67)
Race- Black/African American±	−0.24 (3.81)	0.950 (−7.75, 7.27)	−0.96 (1.82)	0.598 (−4.54, 2.62)	−0.18 (3.04)	0.954 (−6.16, 5.81)	−0.66 (3.02)	0.828 (−6.62, 5.30)
Race - Hispanic ±	−3.35 (4.84)	0.489 (l2.89, 6.19)	−1.86 (2.30)	0.422 (−6.40, 2.69)	2.78 (3.85)	0.471 (−4.81, 10.38)	−6.27 (3.84)	0.104 (−13.84, 1.29)
Race - Other±	−2.27 (4.95)	0.647 (−12.03, 7.48)	2.06 (2.36)	0.384 (−2.59, 6.70)	5.37 (3.94)	0.175 (−2.41, 13. 14)	1.23 (3.92)	0.755 (−6.51, 8.97)
Intercept	5.91 (8.67)	0.49 (11.18, 23.01)	2.75 (4.13)	0.507 (−5.40, 10.90)	3.10 (6.91)	0.654 (−10.52, 16.72)	−8.56 (6.88)	0.215 (−22 12, 5.00)

*Reference condition: Education – Graduate School

^†^Reference condition: Stage of Decision-making – Decided, unlikely to change mind

±Reference condition: Caucasian

### Decisional self efficacy and decisional conflict outcomes

PDA users, as compared to UC, had substantially larger likelihood of improvements in both decisional self-efficacy (3.7 vs−3.9, respectively) and decisional conflict (−22.2 vs−7.5, respectively) within 6 weeks of enrollment, differences which were highly statistically significant (*p* < .0001). The mean DCS score among PDA users at final follow-up was well under 25 (23.6 [sd 14.3]). Significant improvements in each of the decisional conflict scale sub-scores were observed (Table 3).

Multivariate analyses adjusting for educational, race, stage of decision-making, importance of cost in making medication decisions, previous yeast or urinary tract infections, and other factors found that PDA use was associated with a substantial likelihood of decline in decisional conflict (−16.25 (*p* < 0.0001)) and improvement in decisional self-efficacy ( 7.44 [*p* = 0.004]) (Table 4).

Among those assigned to the intervention group, 95 (83.3 %) responded to a brief email survey shortly after viewing the PDA. 94 (98.9 %) reported the information was “just right” and “well balanced” and 90 % that the information was sufficient to understand each class of antihyperglycemic agent’s effectiveness and side effects. Twelve (12.6 %) found the length of the material “too long”. Ten clinicians who enrolled a minimum of ten subjects were asked to re-review the PDA at the end of the study and answer (a) similar question. Of the nine who responded, six (66.6 %) endorsed the statement that the PDA “could help patients to fully understand the risks and benefits of treatment options for T2DM” either “a great deal” or “quite a bit”, with the remainder (33.3 %) responding “at least somewhat”.

## Discussion

Engaging patients and their families as active participants and collaborators in care is critical for achieving patient centered health care in the U.S., improving the patient experience of care, improving outcomes and reducing the per capita costs of health care [49]. Among people with diabetes, the Diabetes Attitudes, Wishes and Needs (DAWN2) study recently indicated that engagement and participation by people with diabetes is lacking, but is a high priority for both patients and health professionals [50, 51]. In addition, patient engagement is particularly important in the management of high-risk diabetes patients [52]. However, shared decision-making that provides the amount and depth of information targeted to what is important to patients in support of meaningful patient-provider collaboration is time consuming and contributes to delays in the advancement and intensfication of treatment [53, 54]. The development and use of health information using technology platforms, such as this PDA, is effective, acceptable to patients, and has the potential to provide the knowledge and confidence patients need to more fully participate in shared decision-making and become true partners in self-management and their future health. To support communication and engagement in decision making, we developed and tested the Diabetes Decision Aid for T2DM. This decision aid is the first for T2DM widely available in an electronic format that not only provides targeted information on medication choices, but also facilitates users’ understanding of personal values related to important aspects of the medication decision through a values a clarification exercise, including each option’s risks and benefits. Values clarification help patients understand and recognize if treatment decision are consistent with the priorities identified in the exercise. Helping patients make explicit value assumptions may support decision-making processes with fuller understanding and buy in, which is integral to one’s self-confidence or belief in the ability to make decisions (decision self-efficacy) [55, 56].

PDA use was associated with substantial and significant improvements in knowledge about T2DM medication, decisional self-efficacy, and decisional conflict in a large socio-demographically diverse sample. We discovered several apparent contradictions. Even though all subjects had been advised by their provider to consider additional antihyperglycemic medication, more than half of the subjects in both groups reported that they had not begun to consider their treatment options. Most (70 %) participants preferred an active role in decision-making, yet few had adequate knowledge to make a good decision. This suggests a substantial unmet need to both activate patients to engage in decision-making and to help them improve their knowledge about T2DM and its treatment.

### Knowledge

The gains in knowledge that we observed in our diverse national sample (approximately 23 points on a normalized scale) are consistent with findings from other PDAs attempting to improve knowledge about diabetes antihyperglycemic medication options in different settings. Branda et al. reported knowledge gains of 23.5 [95 % CI 9.7, 37.3] among a largely Caucasian sample of T2DM patients in rural Minnesota [35]. In this study, the patients receiving the Diabetes Choice PDA achieved a final correct score of 57 % and the control group achieved a correct score of 33 %, similar to our findings of 55.5 and 32.5 % correct, respectively. Both studies demonstrated that although gains in knowledge and knowledge confidence were substantial in the PDA groups. The Statin Choice Trial [28], conducted among patients with diabetes exploring adding statins to their medication regimens, found that their PDA improved knowledge of medication risks and benefits by approximately 27 points in a highly educated Caucasian population in Minnesota, administered during a face-to-face consultation. The PANDAs PDA [34] included predominantly well-educated Caucasians in the UK and reported mixed results for knowledge when two questions were asked to assess understanding of medication effectiveness and risks. Our study provides further evidence of a substantial gap in knowledge about T2DM and its treatment in an educationally and racially/ethnically diverse national sample. Our findings support the need for better targeted patient education about T2DM and antihyperglycemic treatments to help patients prepare for shared decision-making [52].

### Decsional self efficacy

Improvements in decisional self-efficacy were found in the PDA group. This may have been due to the positive relationship between self-reported efficacy expectations and knowledge acquisition [15, 57]. An unexpected outcome was a decrease in decisional self-efficacy in the UC group. When people are confronted with a challenging task, some may become less sure of their efficacy [58]. Baseline decisional self-efficacy was measured prior to administering a detailed knowledge test and a questionnaire addressing the many factors that must be considered when selecting medication (e.g., minimizing the risk of hypoglycemia, adverse events). Subjects may have overestimated their self-efficacy at baseline [53], and UC self-efficacy may have been negatively impacted upon recognizing how little subjects actually knew about their medication options and how many factors needed to be considered. To our knowledge, this study is the first to observe a decrease in self-efficacy in the control arm of studies of treatment-focused patient decision aids. Of 115 studies in the current Cochrane systematic review, 8 measured the effect of decision aids on confidence when comparing decision aid use to usual care, 3 of which used decisional self-efficacy as that measure of confidence. None of these studies measured outcomes related to a decision aid for diabetes and only one measured change in DSES from baseline. Atterbern et al. [59] randomized subjects to a video PDA vs written materials for information regarding bariatric surgery. No decreases in self-efficacy were observed in the control group receiving the pamphlet, and no significant differences in DSES change from baseline were observed in the treatment group versus the control group.

### Decisional conflict

The PDA significantly lowered overall decisional conflict and each of its subscales. Mean DCS scores at final follow-up were <25, the threshold below that equates to making an effective decision [43]. Some of these findings were unexpected because the PDA did not explicitly target some of the domains measured by the subscale, such as feeling more supported in their decision-making. Only six of 15 treatment-related PDAs in the most recent Cochrane review reported significant findings for this subscale [26]. These improvements in decisional conflict subscales may reflect unique features of our PDA, such as tailoring information according to the patients’ preferences (e.g., expected degree of glycemic control, and durability of control; impact on weight; risk of hypoglycemia; costs). This type of tailoring might make patients feel more supported in their choice. The PDA also includes interactive values clarification exercises that help patients weigh relative the importance of each of these values, which is different than the more common static printed or videotaped PDA material. These exercises may explain our positive impact on the values clarity subscale.

### Limitations

Our study has several limitations. First, our blinding of referring clinicians prevented us from engaging providers in shared decision-making training, which has been shown to augment the effect of PDAs [60]. Thus our findings may underestimate the impact of our PDA where provider shared decision-making training is included. On the other hand, subjects were not blinded to treatment assignment, and this may have impacted results due to expectations raised regarding PDA participation benefits. Also, because viewing the PDA and assessments were conducted over the internet, results may not be generalizable to a less-internet experienced audience, or to an audience with socioeconomic barriers to internet access. Our choice of a comparator, usual care, is not an ideal comparison for PDA assessments. An alternative may have been to randomize control subjects to a time-matched subject information page. This would help assure that differences between groups was related to PDA content, and not the system of support inherent and facilitated by the PDA delivery system. Our sample size was relatively small, thus larger studies are warranted. Although we enrolled a diverse group of subjects, we did not directly measure literacy or numeracy but instead used educational level as a proxy.

## Conclusions

This study provides insights into the impact of a novel interactive PDA for subjects with T2DM for whom first line treatment with metformin is no longer effective. The PDA helps patients acquire essential knowledge to make informed decisions according to their personal values and preferences. It substantially and significantly improved knowledge, decisional self-efficacy, and decisional conflict among a diverse group of patients with T2DM. We developed a Spanish translation of the PDA for a Latino/Hispanic audience, but have not yet tested it. It would be important to understand its impact in Spanish-speaking populations. The PDA is currently available and being deployed in a variety of US health systems (www.diabetesdecisionaid.com. Future studies should assess the impact of PDA on medication choice, medication adherence, patient-provider communication, glycemic control, other clinical outcomes, and costs.

### Ethics apprproval and consent to participate

All study procedures, including informed consent were approved by the New England IRB (#14-104).

### Consent for publication

Not applicable.

### Availability of data and materials

Data and materials are available upon written request to the corresponding author.

## Additional file

Additional file 1:
**T2DM Medication Knowledge Questions.** (DOCX 16 kb)

## Abbreviations

DAWN2: Diabetes attitudes, wishes and needs (DAWN2) STUDY
DCS: Decisional conflict scale
DPP-4: Dipeptidyl peptidase-4
DSES: Decisional self efficacy scale
GLM: Generalized Linear Model
GLP-1: Glucagon-like peptide-1 (GLP-1S) agonists
ITT: Intent to treat
MCMC: Markov-Chain-Monte-Carlo
MO: Missouri
PDA: Patient decision aid
SAS: Statistics analytics system
SD: Standard deviation
SGLT-2: Sodium-glucose co-transporter 2 inhibitors
T2DM: Type 2 diabetes mellitus
TZD: Thiazolidinediones
UC: Usual care
US: United States
