# Supplementary material for: Effect of a patient decision aid (PDA) for type 2 diabetes on knowledge, decisional self-efficacy, and decisional conflict
Source: BMC Health Serv Res. 2016 Jan 14;16:10. doi: 10.1186/s12913-016-1262-4 (PMC4712511; doi:10.1186/s12913-016-1262-4)
Supplement: Additional file 1: — T2DM Medication Knowledge Questions. (DOCX 16 kb) [file 12913_2016_1262_MOESM1_ESM.docx]

**Appendix -** T2DM Medication Knowledge Questions

Currently available options that can be added to metformin to help lower my blood sugar include:

•Sulfonylureas

•DPP-4s

•Statins*

•TZDs

•SLGT-2s

•Beta blockers*

•GLP-1s

•Insulin

Some medications that can be added to metformin do a pretty good job at helping people lose weight.

Some medications that can be added to metformin do a pretty good job at helping people not gain weight.

Some medications that can be added to metformin might make people gain weight.

Only medications that are given by injection increase risk of having hypoglycemia or “lows”.*

There are some medications that can be added to metformin that should only be given to someone with a healthy heart.

Insulin is the only class of medicine for diabetes that is taken as a shot (injection).*

The medicines that work best for most people to lower blood sugar are usually taken more than once a day.*

Medications that are taken as a shot (injection) are almost always better at lowering blood sugar compared to pills.*

The oral medications that can be added to metformin all lower blood sugar about the same amount.*

*False statements

| Participating Clinicians | | |
| --- | --- | --- |
| Abraham, Benjamin, MD | Snellville | GA |
| Anderson, Charles, MD | San Marcos | TX |
| Anderson, Chrystal, MD | Indianapolis | IN |
| Barrington, Patricia, MD | Snellville | GA |
| Bretton, Elizabeth, MD | Albuquerque | NM |
| Center for Preventative Medicine (Lucas, Stephanie, MD) | Cross Point | MI |
| Choudhury, Rahul, MD | Jellico | TN |
| Clark, Gordon, MD | Shawnee | KS |
| Covenant Community Care (Abbo, Marissa, MD) | Detroit | MI |
| Devu, Indira, MD | Marietta | GA |
| Ellis, Mark | Enterprise | AL |
| Ellison, Howard, MD | Conyers | GA |
| Fields, Carolyn, MD | Greenville | SC |
| Marshall, Eric, MD | Washington | DC |
| Mazhar, Salma, MA | Mesquite | TX |
| McCool, James, MD | Altamonte Springs | FL |
| Merkes, Kevin, MD | Poway | CA |
| Nguyen, Thy | Baltimore | MD |
| Nova Medical and Urgent Care (Dondlinger, Graham, MD) | Ashburn | VA |
| Octain, Bikan, MD | Annandale | VA |
| Pangtay, Dennis, MD | Irving | TX |
| Pothiwala, Yakub, MD | Tampa | FL |
| Raikhel, Marina, MD | Lomita | CA |
| Ramayya, Aruna, MD | Rancho Palos Verdes | CA |
| Sims, James, MD | Bellaire | TX |
| VanWingen, Jeffery, MD | Grand Rapids | MI |
| Whitmer, Daniel, MD | Beavercreek | OH |
